# Upregulation of Heme Oxygenase-1 Combined with Increased Adiponectin Lowers Blood Pressure in Diabetic Spontaneously Hypertensive Rats through a Reduction in Endothelial Cell Dysfunction, Apoptosis and Oxidative Stress

**DOI:** 10.3390/ijms9122388

**Published:** 2008-12-01

**Authors:** Jian Cao, George Drummond, Kazuyoshi Inoue, Komal Sodhi, Xiao Ying Li, Shinji Omura

**Affiliations:** 1 Department of Pharmacology, New York Medical College, Valhalla, NY 10595 USA. E-Mails: gdrummond@infacare.com (G. D.); Kazuyoshi_Inoue@nymc.edu (K. I.); Komal_Sodhi@nymc.edu (K. S.); Shinji_Omura@nymc.edu (S. O.); 2 Department of Geriatric Cardiology, Chinese PLA General Hospital, Beijing 100853 China. E-Mail: calvin301@163.com (J. C.); lixy@mx.cei.gov.cn (X. L.)

**Keywords:** Heme oxygenase, hypertension, diabetes, adiponectin, oxidative stress, apoptosis

## Abstract

This study was designed to investigate the effect of increased levels of HO-1 on hypertension exacerbated by diabetes. Diabetic spontaneously hypertensive rat (SHR) and WKY (control) animals were treated with streptozotocin (STZ) to induce diabetes and stannous chloride (SnCl_2_) to upregulate HO-1. Treatment with SnCl_2_ not only attenuated the increase of blood pressure (p<0.01), but also increased HO-1 protein content, HO activity and plasma adiponectin levels, decreased the levels of superoxide and 3-nitrotyrosine (NT), respectively. Reduction in oxidative stress resulted in the increased expression of Bcl-2 and AKT with a concomitant reduction in circulating endothelial cells (CEC) in the peripheral blood (p<0.005) and an improvement of femoral reactivity (response to acetylcholine). Thus induction of HO-1 accompanied with increased plasma adiponectin levels in diabetic hypertensive rats alters the phenotype through a reduction in oxidative stress, thereby permitting endothelial cells to maintain an anti-apoptotic environment and the restoration of endothelial responses thus preventing hypertension.

## 1. Introduction

Cardiovascular and renal disease are the leading causes of morbidity and mortality in patients with diabetes [1]. Hypertension is an independent risk factor for both macrovascular (stroke, myocardial infarct, peripheral vascular disease) and microvascular (nephropathy, neuropathy, retinopathy) complications and is a common co-existing condition in diabetes [1]. Oxidative stress has been implicated in the pathogenesis of insulin resistance, type 2 diabetes, and its cardiovascular complications [2, 3]. Excessive generation of reactive oxygen species (ROS) in diabetes is the underlying mechanism of endothelial injury, resulting in an accelerated rate of apoptosis and endothelial cell sloughing [4,5]. Oxidative stress underlies hypertension in many animal models [6]. Superoxide (O_2_^−^) raises blood pressure (BP) by both central [7] and peripheral mechanisms [8]. Recent studies indicate that the combination of diabetes and hypertension adversely affects oxidative stress in the kidney [9, 10], this is associated with the high prevalence of chronic kidney disease. In the 1999 to 2000 National Health and Nutrition Examination Survey (NHANES), 40.4% of people with diabetes had hypertension [11]. In patients with newly diagnosed diabetes, hypertension is associated with a 56% increased risk for cardiovascular morbidity and mortality [12]. Even prehypertension, which is a more prevalent (59.4% vs. 48.2%, P <0.001) in patients with diabetes, confers additional cardiovascular risk. Compared with controls, in patients with diabetes with and without prehypertension, the hazard ratios for developing cardiovascular disease are 3.70 and 2.90, respectively [13]. One central therapeutic approaches to prevent diabetic and hypertensive organ damage is strict blood glucose control, however blood pressure must also be rigorously controlled [14]. Therefore, in order to develop a drug with therapeutic efficacy as an antihypertension and antidiabetes agent is of great importance that it should have renoprotective properties.

The heme oxygenase (HO) system displays both antioxidant and anti-apoptotic properties because of its degradation products, bilirubin/biliverdin and carbon monoxide (CO), respectively [15]. The beneficial effects of upregulation of HO-1 in antihypertension, antidiabetes and renoprotection have been reported in a series animal models [15]. The HO system has been implicated in the regulation of blood pressure and the blood pressure lowering effect of HO-1 induction has been attributed to various mechanisms, including decreased production of vasoconstrictor eicosanoids and increased production of CO [15]. CO is a vasodilator, a stimulator of soluble guanylate cyclase, an endogenous modulator of the cGMP signaling system, an activator of calcium-activated potassium channels (KCa) in vascular smooth muscle and an inhibitor of endothelin-1 mediated vasoconstriction [15–17]. HO has been reported to be involved in the regulation of renal salt excretion. CO generated by HO-dependent heme catabolism stimulates the apical 70-pS K-channel [18]. Inhibition of HO activity decreases sodium and fluid reabsorption in the thick ascending limb of the loop of Henle in the rat [19]. Furthermore, exogenous heme administration induces HO-dependent natriuresis and diuresis which were blocked by SnMP pretreatment [20]. These reports reinforce the potential importance of HO in the regulation of kidney function. However, no study has examined the role of HO-1 in an animal model with both hypertension and diabetes.

The objective of this study was to examine HO-1 induction in relation to the renal HO system, plasma adiponectin levels, endothelial function, the extent of endothelial cell sloughing, oxidant levels and blood pressure in a diabetic hypertensive animal model (SHR). Our results demonstrate that basal levels of the constitutively expressed HO-2 are not increased, but O_2_^−^ and nitrotyrosine levels were increased in SHR compared to Wistar-Kyoto (WKY) rats. Administration of stannous chloride (SnCl_2_), a potent inducer of HO-1 protein and HO activity, attenuated the development of hypertension. This was associated with the elevation of plasma adiponectin levels, decreased endothelial apoptosis, oxidative and nitrosative stress and increased production of CO and bilirubin, thereby demonstrating a significant role for HO-1 in cellular defense against endothelial dysfunction, oxidant damage and the maintenance of renal hemodynamic function in the diabetic hypertensive animal model. These findings have important clinical implications in the management of hypertension in the diabetic patients.

## 2. Results and Discussion

This is the first study suggests the existence of an HO-1-adiponectin axis that is central to lowering blood pressure in diabetic SHRs. In the present study, increased renal HO-1 expression and HO activity with a concomitant increase in plasma adiponectin levels resulted in a reduction in the level of hypertension as manifest by a decrease in blood pressure in a diabetic hypertensive animal model. In addition, the diabetes-mediated increase in contractility in femoral arteries and CECs in peripheral blood was reversed by increase in HO-1 expression and HO activity. The upregulation of HO-1 was associated with a concomitant decrease in the levels of O_2_^−^ and 3-NT, markers for oxidative stress. In addition, there was an increase in the expression of the anti-apoptotic proteins, Bcl-2 and AKT. Thus, it appears that the induction of renal HO-1 combined with increased plasma adiponectin levels in the diabtetic SHR animal model results in a change of phenotype from pro- to anti-apoptotic.

### 2.1. Effect of SnCl_2_ on HO-1 and HO-2 expression in SHR and WKY rats

**Figure 1.**
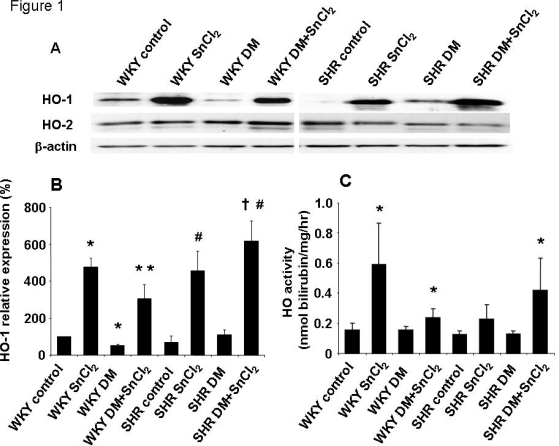
A) Western blot and densitometry analysis of HO-1 and HO-2 proteins in kidney from WKY rats or SHRs. Rats were treated with SnCl_2_ (once a week 50 mg/kg body weight for 4 weeks, S.C.), STZ (once a week 65 mg/kg body weight for 4 weeks i.v. for diabetic models) or both SnCl_2_ and STZ. Immunoblots were performed with antibodies against rat HO-1 and HO-2. Data are representative of 4 separate experiments. B) Mean band density normalized relative to β-actin (n=4, * p<0.05 vs. WKY control rats, * * p<0.05 vs. WKY DM (diabetic models), # p<0.05 SHR control, ^†^ p<0.05 vs. SHR DM). C) HO activity in kidney from WKY rats or SHRs (n=4, * p<0.05 vs. corresponding control rats).

Figures 1A–C depict the inducing effect of SnCl_2_ (50 mg/kg bw s.c.), as manifest by an increase in the levels of renal HO-1 protein in both SHR and WKY rats (Figures 1A and B). In addition, we examined the effect of diabetes on the expression of HO-1 and HO-2 in rat kidneys. As seen in Figures 1A and B, SnCl_2_ caused significant increases in the levels of HO-1 protein, but not in HO-2 protein in both SHR and WKY rats. Furthermore, HO activity increased from 0.16 nmol/hr/mg protein in the kidneys of WKY animals to 0.24 nmol/hr/mg protein in SnCl_2_-treated animals and from 0.13 nmol/hr/mg protein in the kidneys of diabetic SHR to 0.42 nmol/hr/mg protein in SnCl_2_-treated animals after four days of administration (Figure 1C).

### 2.2. Effect of HO inducers on COX-2 in the diabetic kidney

HO-1 induction is associated with a reciprocal decrease in arachidonic acid metabolism by cytochrome P450 (CYP450) monoxgenases and cyclooxygenases (COX) and to a decrease in blood pressure in SHR, therefore we measured the expression of COX-2 in the kidneys of SHR and WKY rats. Western blot analysis revealed significantly (p<0.05) higher levels of COX-2 protein expression in both diabetic WKY and SHR animals compared to control rats (data not shown). SnCl_2_ administration resulted in a decrease in the levels of COX-2 expression in the kidneys of both animal groups. In both the WKY and SHR SnCl_2_-treated animals, the levels of COX-2 expression returned to control levels (data not shown). SnCl_2_ administration had no effect on COX-2 expression in nondiabetic animals.

### 2.3. Effect of HO-1 induction on plasma adiponectin levels

**Figure 2.**
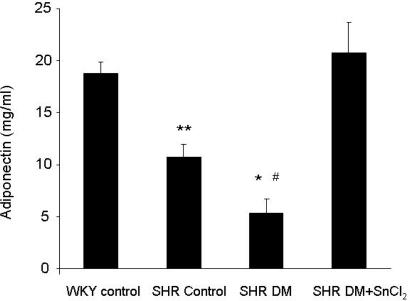
Plasma adiponectin levels of WKY rats and SHRs (* p<0.05 vs. WKY control and SHR control respectively, # p<0.01 vs. WKY control and SHR DM + SnCl_2_ respectively, ** p<0.05 vs. WKY control and SHR DM + SnCl_2_, respectively).

To understand the possible mechanism by which HO-1 mediated increase in antioxidants levels, we measured adiponectin levels. There was a significant decrease in plasma adiponectin levels in diabetic SHRs compared to SHR controls (Figure 2). SnCl_2_ administration resulted in a marked increase in the levels of plasma adiponectin in diabetic SHRs as compared to diabetic untreated animals (Figure 2). Moreover, plasma adiponectin in diabetic SHRs attained levels similar to those in the WKY control animals (Figure 2).

### 2.4. Effect of HO-1 induction on O_2_^−^ production in the kidney

**Figure 3.**
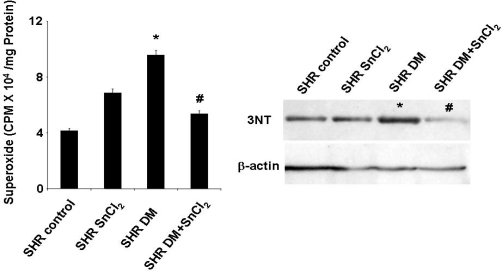
O_2_^−^ production levels of kidney and western blot analysis of nitrotyrosine in SHRs (* p<0.05 vs. SHR control, # p<0.05 vs. SHR DM).

O_2_^−^ production was assessed in the kidneys of SHR animals using a low concentration of lucigenin (5 μm). As seen in Figure 3, kidneys obtained from diabetic SHR show a significant increase in O_2_^−^. SnCl_2_ administration resulted in a decrease in O_2_^−^ levels in SHR control animals. This decrease paralleled the decrease in COX-2 levels but was the reciprocal of the increase in HO activity and HO-1 levels described above (Figure 1 A–C).

### 2.5. Effect of HO inducers on nitrotyrosine levels

Nitrotyrosine (3-NT) levels were measured using Western blot analysis and untreated diabetic rats showed increased levels of 3-NT. Treatment of diabetic animals with SnCl_2_ resulted in a decrease in 3-NT to levels lower than those in controls (Figure 3). SnCl_2_ administration had no effect on 3-NT levels in nondiabetic SHR. (Figure 3).

### 2.6. Effect of HO expression on the development of hypertension

**Figure 4.**
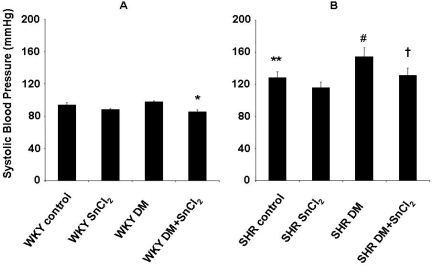
A and B. Blood pressure was measured by the tail cuff method after the treatment (* p<0.05 vs. WKY DM, ** p<0.05 vs. WKY control, # p<0.05 vs. SHR control, ^†^ p < 0.05 vs. SHR DM).

Systolic blood pressure was significantly (p<0.05) increased in SHR compared to WKY rats. In diabetic WKY rats, SnCl_2_ treatment resulted in a small but significant (p<0.05) decrease in blood pressure compared to controls (Figure 4). In the untreated diabetic SHR, systolic blood pressure was significantly higher than that of the SHR controls (154 ± 11 mmHg vs. 128 ± 8 mmHg, p<0.05) (Figure 4). Treatment with SnCl_2_ resulted in a decrease in systolic blood pressure in diabetic SHR to levels of the SHR controls (Figure 4).

### 2.7. Effect of HO-1 expression on vascular responses to PE and Ach

**Figure 5.**
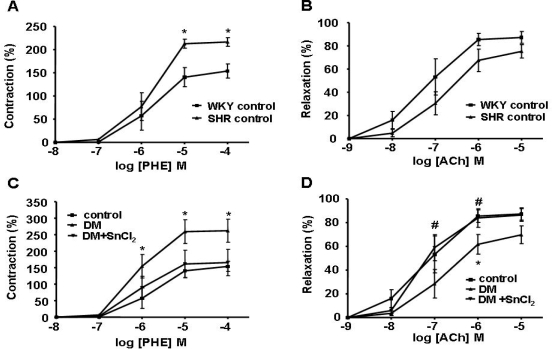
A) The ring segments from femoral arteries were exposed to phenylephrine (PE) in a dose-dependent manner (10^−8^ − 10^−4^ M). PE-induced contraction of SHRs was significantly increased as compared to WKY rats (*p<0.05 vs. WKY control rats). B) Femoral arteries were precontracted with phenylephrine (PE) and then exposed to acetylcholine (Ach) in a dose dependent manner (10^−9^ − 10^−5^ M). Vessels from SHRs demonstrated a decreased response compared to WKY rats. C) The ring segments from femoral arteries were exposed to phenylephrine (PE) in a dose-dependent manner (10^−8^ − 10^−4^ M). PE-induced contraction of WKY rats treated with STZ was significantly increased as compared to control rats, this increase was fully reversed in WKY rats treated with STZ and SnCl_2_ (*p<0.05 vs. WKY control rats). D) Femoral arteries were precontracted with phenylephrine (PE) and then exposed to acetylcholine (Ach) in a dose dependent manner (10^−9^ − 10^−5^ M). Vessels from WKY rats treated with STZ demonstrated a decreased response compared to control rats significantly, which was fully reversed by SnCl_2_ treatment (*p<0.05 vs. WKY control rats, # p<0.05 vs.WKY DM rats).

**Figure 6.**
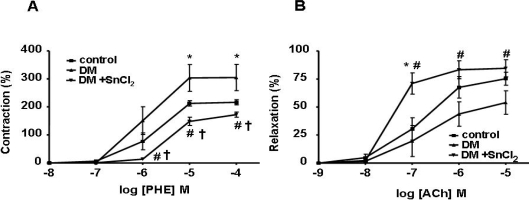
A) The ring segments from femoral arteries were exposed to phenylephrine (PE) in a dose-dependent manner (10^−8^ − 10^−4^ M). PE-induced contraction of SHRs treated with STZ was significantly increased as compared to control rats. With SnCl_2_ administration, the contraction in SHRs treated with STZ was more decreased compared to control rats (*p, ^†^p<0.05 vs. SHR control, #p<0.05 vs. DM rats). B) Femoral arteries were precontracted with phenylephrine (PE) and then exposed to acetylcholine (Ach) in a dose dependent manner (10^−9^ − 10^−5^ M). Vessels from SHRs treated with STZ demonstrated a decreased response compared to control rats significantly, which was reversed by SnCl_2_ administration (*p<0.05 vs. SHR control, #p<0.05 vs. DM rats).

To assess the effect of HO-1 expression on vascular response, we measured PE-induced contraction and ACh-induced relaxation in the femoral artery. A significant increase in contraction and impairment of relaxation was observed in SHR compared to normal WKY rats (Figures 5A and B, respectively). SnCl_2_ treatment restored vascular reactivity in diabetic WKY rats to normal levels (Figures 5C and 5D). In addition, not only was the contraction in treated diabetic SHR decreased (Figure 6A) but the relaxation was increased compared to untreated rats (Figure 6B), suggesting the existence of endothelial cell damage in hypertensive rats and that HO-1 induction not only restores but also improves endothelial dysfunction.

### 2.8. Endothelial cell sloughing

**Figure 7.**
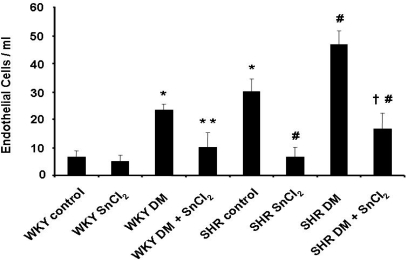
Number of circulating endothelial cells in control and diabetic rats was measured. Number of cells in SHR controls increased significantly compare to WKY control rats. STZ treatment increased the cells significantly in WKY rats and SHRs relative to controls. Administration of SnCl_2_ decreased circulating endothelial cells in WKY DM rats, SHR control and SHR DM, but had no significant effect in WKY control rats (*p<0.05 vs. WKY control rats, * * p<0.05 vs. WKY DM rats, #p < 0.05 vs. SHR control rats, ^†^p < 0.05 vs. SHR DM).

Because endothelial cell sloughing is related to endothelial damage, CEC were isolated from peripheral blood and counted. Cells were counted if they had at least 10 immunomagnetic beads attached, fluoresced under ultraviolet light after staining with acridine orange, and maintained the round to oval shape and the 20–50 μm size typical of endothelial cells. As seen in Figure 7, the number of CEC was significantly higher in diabetic animals compared to controls. Treatment with SnCl_2_ reduced the number of CEC in both diabetic SHR and WKY animals, reflecting the existence of endothelial damage in hypertensive animals (Figure 7).

### 2.9. Effect of HO-1 induction on Bcl-2 in SHR and WKY rats

**Figure 8.**
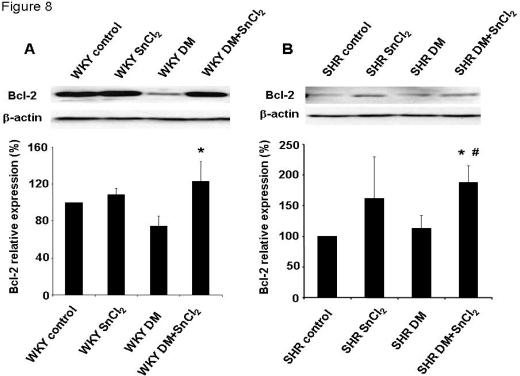
A and B. Western blot and densitometry analysis of anti-apoptotic Bcl-2 in kidney from WKY rats (A, * P<0.05 vs. WKY DM rats) or SHRs (B, * p<0.05 vs. SHR control, #p<0.05 vs. SHR DM). Mean band density normalized relative to β-actin.

We examined the levels of Bcl-2, an anti-apoptotic protein, since oxidative stress results in the activation of multiple signaling cascades that ultimately dictate the outcome for cell survival. As seen in Figures 8A and B, Bcl-2 expression was significantly decreased in diabetic animals compared to controls. Treatment with SnCl_2_ restored the levels of Bcl-2 in both diabetic SHR and WKY animals. A similar response was seen with AKT (results not shown).

The dramatic change in phenotype described above was highlighted by the decrease in the number of CEC in the peripheral blood of SnCl_2_-treated diabetic animals when compared to control diabetic animals. Diabetes increases contractility in femoral arteries; however, in the present study, contractility was reversed by the induction of renal HO-1. Indeed, in diabetic animals, HO-1 induction resulted in the restoration of contractility to pre-diabetic levels in both SHR and WKY animals. Increased relaxation to Ach after CoPP treatment has been reported using the femoral artery model [21]. CoPP, like SnCl_2_, is a powerful inducer of HO-1. Increased relaxation has also been achieved by the use of the CO-releasing molecule CORM-3, but not with the antioxidant heme-degradation product, biliverdin [21]. Recently, we reported that HO-1 upregulation by CoPP prevents the diabetic state in non-obese diabetic (NOD) mice [22]. Also, exogenously administered CO and bilirubin both can prevent endothelial cell sloughing in diabetic rats, presumably via a decrease in oxidative stress, and thus represent a novel approach to prophylactic vascular protection in diabetes [23]. To examine the impact of HO-1 expression on the vascular system, PE-induced contraction and Ach-induced relaxation were measured in the femoral artery. A significant increase in contraction and impairment of relaxation was observed in hypertensive animals when compared to WKY control animals (Figure 5). The administration of SnCl_2_ to hypertensive animals resulted in both a decrease in contraction and an increase in relaxation compared to untreated animals (Figure 6). Thus, it appears that increased renal HO-1 expression not only restores endothelial cell function but also improves pre-existing endothelial damage in the SHR animal model of diabetes. Indicating the seminal role of HO-1 in both preventing and reversing diabetes-induced endothelial dysfunction in this animal model. Thus, the use of HO inducers may be of clinical benefit in individuals with significant endothelial cell dysfunction in an attempt to correct existing clinical deficits. Also, the beneficial effects of HO-1 overexpression on blood glucose in this animal model of diabetes encourages the development of this approach in the clinical arena.

Hyperglycemia-mediated increases in O_2_^−^ formation and ROS contribute to endothelial and beta cell apoptosis and dysfunction. These defects may be reversed by the overexpression of antioxidant enzymes and the administration of antioxidants. In the present study, overexpression of HO-1 resulted in decreased O_2_^−^ generation, which may be due to a decrease in the levels of NADPH oxidase [24], a heme-dependent protein, and/or an increase in the levels of EC-SOD [25]. In addition, the heme degradation products, CO and bilirubin, have, respectively, potent anti-inflammatory and anti-apoptotic activities and antioxidant properties [15]. Thus, the induction of HO-1 appear to provide a favorable cellular environment for survival that is rich in antioxidants. Indeed, HO-1 overexpression results in a phenotype that is both anti-oxidative and anti-apoptotic. Our results show that generation of O_2_^−^ was prevented by increased renal HO-1 expression in diabetic SHRs. This was associated with concomitant decreases in O_2_^−^, 3-NT and COX-2 expression and increases in Bcl-2 and AKT expression.

Renal medullary HO activity plays an essential role in the control of pressure natriuresis and arterial blood pressure and that impairment of this HO/CO-mediated antihypertensive mechanism results in the development of hypertension [26]. Kidney-specific induction of HO-1 prevents the development of Ang II-dependent hypertension and that induction of HO-1 in the kidney may be the mechanism by which systemic delivery of HO-1 inducers lowers blood pressure in Ang II-dependent hypertension [27]. 3-nitrotyrosine, cellular heme and superoxide, all promoters of vascular damage, are reduced by HO-1 induction, thereby preserving vascular integrity and protecting renal function through a decrease in blood pressure and an increase in antiapoptotic proteins. In contrast, inhibition of HO activity exacerbated renal damage in a renovascular hypertension animal model [28,29]. Upregulation of HO-1-linked signaling pathways and reversal of vascular remodeling in peripheral blood vessels likely mediate the antihypertensive effect of hemin [30]. Human HO-1 gene transfer lowers blood pressure and promotes growth in spontaneously hypertensive rats [31]. Upregulation of HO-1 prevents the generation of oxidative stress only when the anti-oxidant defense system is operative [32]. Individuals with low levels of HO-1 (caused by HO-1 genetic polymorphism) are more likely to have renal injury and express a hypertensive phenotype following chronic ingestion of low-level Cd, compared with those having higher levels of HO-1 [33]. HO-1 is essential for the antiproliferative and vascular protective effects of rapamycin in vitro and in vivo in monocrotaline-induced pulmonary hypertension [34]. The mesenteric vasodilator action of 11,12-EET mediated vascular relaxation is via an increase in HO activity and activation of calcium-activated potassium channels [35]. CO releasing molecules (CO-RMs) are effective therapeutic agents that deliver CO during kidney cold preservation and can be used to ameliorate vascular activity, energy metabolism and renal function at reperfusion [36]. It has been reported that bilirubin may effect the DOCA-salt model of systemic hypertension [37]. The pressor and pro-oxidant effects of Ang II are attenuated in the hyperbilirubinemic rat, an effect which may reflect scavenging of superoxide anion by bilirubin [38]. In agreement with these results, our previous studies demonstrated higher levels of HO-1 in the renal medulla thus reinforcing the role of the HO/CO system in the regulation of renal medullary blood flow. HO-1 in mTAL plays a vital role in protecting against various noxious stimuli [39,40]. In the glycerol model of acute renal failure, increased release of heme proteins caused renal toxicity whereas pre-induction of HO-1 preserved renal function [41]. The protective actions of HO-1 are not confined to the attenuation of oxidant levels, but extend to amelioration of inflammation, atherosclerosis, transplant pathobiology and ischemia [15].

Abraham’s group found that upregulation of HO-1 can increase adiponectin levels in several animal models [42–46]. The beneficial effects of adiponectin in a variety of cardiovascular diseases have been reviewed [47, 48], especially there is a negative relationship between adiopnectin and hypertension [49]. The mechanism by which HO-1 is involved in increased adiponectin levels is related to the function of HO-1 as a stress response/chaperone protein as well as its ability to decrease ROS by increasing glutathione and EC-SOD levels [5, 15, 25] and by decreasing O_2_^−^ production [42, 50]. PPARγ agonists, are shown to induce both HO-1 [51] and the rate-limiting chaperone protein EroL [52,53]. PPARγ agonist which increases adiponectin may do so by increasing the levels of EroL chaperone protein [52]. Since PPARγ also increases HO-1 protein levels [51] and HO-1 is known as a chaperone protein, it is possible that one of the mechanisms by which HO-1 can increase adiponectin levels is through more efficient adiponectin stabilization and protection. This would confirm the report of Wang et al who showed that the chaperone protein EroL increased adiponectin [53]. We also have previously shown that upregulation of HO-1 protein in diabetic rats provided both cardio- and vascular- protection against ROS [42].

**Figure 9.**
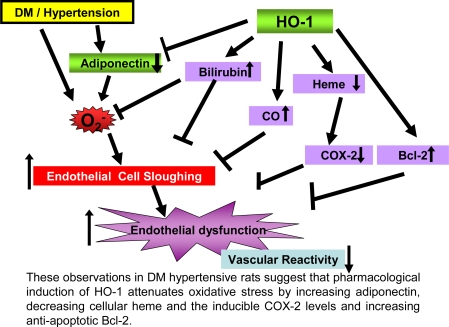
Potential mechanisms underlying the chemoprotective actions of HO-1 in diabetes and hypertension.

Taken together, the results of the present study demonstrate that upregulation of renal HO-1 associated with increased adiponectin can prevent diabetes-induced vascular dysfunction in the SHR model of diabetes. The HO-1-mediated increase in adiponectin resulted in a decrease in blood pressure, a decrease in the number of circulating endothelial cells and restoration of endothelial function in diabetic SHR animals. Indeed, renal HO-1 overexpression can restore pre-existing damaged endothelial cell function. Overexpression of renal HO-1 was associated with a concomitant decrease in the levels of O_2_^−^, 3-NT and COX-2 and an increase in the levels of the anti-apoptotic proteins Bcl-2 and AKT. The clinical significance of these observations can not be overestimated (summarized in Figure 9) as the pharmacological enhancing of HO-1 expression, resulting in increase of HO activity and adiponectin levels, allows the endothelial system to initiate a crucial and immediate host defense against diabetes-mediated perturbations in cellular viability due to increased levels of oxidative stress, thereby preventing the occurrence of high blood pressure and deleterious perturbations in renal function.

## 3. Experimental Section

### 3.1. Animals: induction of diabetes and treatments

Male spontaneously hypertensive rats (SHR) and Wistar-Kyoto (WKY) rats, 180–220 g were used in this study. Diabetes was induced by a single injection of streptozotocin (STZ) (65 mg/kg bw) via the tail vein. STZ was dissolved in 0.05 mol/L sodium citrate buffer (pH 4.5). Age-matched control rats were injected with an equal volume of vehicle (sodium citrate buffer). Rats administered STZ developed hyperglycemia within three days with glucose levels reaching 475±65 mg/dL. To alleviate extreme hyperglycemia and to maintain normal body weight, insulin (NPH 1–3 U/day/100 g bw) was administered three times a week, maintaining glucose levels at 250–300 mg/dL [54]. Glucose was determined (Lifescan, Milpitas, CA) using blood obtained from the tail vain via a capillary tube. All samples were obtained after an overnight fast. Stannous chloride (SnCl_2_) administration commenced the day after diabetes developed. SnCl_2_ (5 mg/100 g bw) was given subcutaneously (s.c.) once a week for four weeks. Systolic blood pressure was measured without anesthesia, using the tail cuff method, at least twice at the baseline and before sacrifice, respectively. Rats were divided into groups as follows: control, STZ, SnCl_2_ and STZ plus SnCl_2_. The Institutional Animal Care and Use Committee approved all experiments, which were conducted under the guidelines for the Care and Use of Laboratory Animals from the National Institutes of Health (NIH).

### 3.2. Tissue preparation

Frozen kidneys were pulverized under liquid nitrogen and placed in a homogenization buffer (10 mM phosphate buffer, 250 mM sucrose, 1 mM EDTA, 0.1 mM PMSF and 0.1% tergitol, pH 7.5). Homogenates were centrifuged at 27,000 g for 10 min at 4°C. The supernatant was isolated and protein levels were determined by the Bradford method. The supernatant was used for measurement of HO-1 and HO-2, COX-2, AKT and Bcl-2 protein and HO activity [43].

### 3.3. Western blot analysis

Protein levels were visualized by immunoblotting with antibodies against rat HO-1 and HO-2 (Stressgen Biotechnologies, Victoria, BC), AKT and Bcl-2 (Cell Signaling Technology, Danvers, MA), COX-2 (Cayman Chemical, Ann Arbor, MI). Briefly, 30 μg of lysate supernatant was separated by 12% SDS/polyacrylamide gel electrophoresis and transferred to Polyvinylidene fluoride or nitrocellulose membrane. Immunoblotting was performed as has been previously described [43]. Chemiluminescence detection was performed with the Amersham ECL or ECL plus detection kit, according to the manufacturer’s instructions (Amersham, Piscataway, NJ).

### 3.4. Measurement of HO activity

HO activity was assayed as previously described [43]. Bilirubin, the end product of heme degradation, is extracted with chloroform and its concentration was determined spectrophotometrically (Perkin-Elmer Dual UV/VIS Beam Spectrophotometer Lambda 25) using the difference in absorbance between λ 460 nm to λ 530 nm with an absorption coefficient of 40 mM^−1^ cm^−1^.

### 3.5. Plasma Adiponectin measurement

The high molecular weight (HMW) form of Adiponectin was determined using an ELISA assay (Pierce Biotechnology Inc., Woburn, MA) as described previously [43,44].

### 3.6. Measurement of vascular activity

Femoral arteries were isolated and connective tissue was removed. Rings measuring 2 mm in length were made from the arteries and incubated in individually thermostated (37 °C) DMT myograph baths (DMT, Atlanta, GA) with a passive tension of 1 g for 1 hour in Krebs bicarbonate buffer (pH 7.4) containing the following (mM): 118 NaCl, 4.7 KCl, 1.5 CaCl_2_, 25 NaHCO_3_, 1.1 MgSO_4_, 1.2 KH_2_PO_4_, and 5.6 glucose, gassed with 21% O_2_−5%CO_2_ (balance N_2_). Force was recorded from force displacement transducers via AD Instrument’s Powerlab system, running Chart 5 software. After I hour of equilibration, we calculated, using a cumulative curve, the concentration of phenylephrine (PE) that determined 70% contraction. Contracted vessels were then given increasing doses of acetylcholine (Ach) (1 nM-10 μM).

### 3.7. Measurement of O_2_^−^ production in the kidney

Previously described methods [55] were used. Homogenized kidney samples of SHRs were placed in plastic scintillation minivials containing 5 μm lucigenin for detection of O_2_^−^ and other additions, in a final volume of 1 ml of air-equilibrated Krebs solution buffered with 10 mmol/L HEPES-NaOH (pH 7.4). Lucigenin chemiluminescence was measured in a liquid scintillation counter (LS6000lC, Beckman Coulter, Inc, Fullerton, CA) at 37°C and data reported as counts/min per milligram of protein after background subtraction.

### 3.8. Detection and quantification of circulating endothelial cells

For immunomagnetic isolation and quantification of circulating endothelial cells (CEC), we used monodispersed magnetizable particles (Dynabeads CELLection Pan Mouse IgG kit) obtained from Invitrogen (Carlsbad, CA). The 4.5 μm diameter polystyrene beads were coated with affinity-purified pan-anti-mouse immunoglobin G1 covalently bound to the surface. The beads were washed, according to the instructions provided by the manufacturer, with a strong magnet (MPC6, Dynal) used to remove sodium azide. Typically, 100 μL of bead suspension was coated noncovalently with 10 μg/mL RECA-1 (Novus Biologicals, Littleton, CO), a pan-rat EL-specific monoclonal antibody diluted 1:10 in PBS-BSA (0.1%) by overnight incubation at 4°C with head-over-head agitation. After three washes with PBS-BSA to remove excess antibodies, the beads were resuspended in buffer until use. RECA-uncovered particles were used as a negative control. If the beads were stored for an extended period of time, 0.1% sodium azide was added to the buffer. Beads and target cells were incubated for 1.5 hours at 4°C on a rotator. The amount of beads (4×10^8^/ml) was in great excess of target cells (>2,000 beads per target cell). Separation of beads and rosetted cells from the blood samples required a minimum exposure of 1 minute to the magnet. Three washes were performed to completely remove nonrosetted cells. After the third wash, rosetted cells were recovered in a solution of acridine orange (a viral fluorescent dye at a final concentration of 5μg/mL in PBS, 150 μL) and subjected to fluorescence microscopy (Olympus 1X81 F).

### 3.9. Statistical analysis

Data are presented as mean±SEM for the number of experiments. Statistical significance (p<0.05) between experimental groups was determined by the Fisher method of analysis of multiple comparisons. For comparison between treatment groups, the null hypothesis was tested by a single-factor ANOVA for multiple groups or unpaired *t* test for two groups.

## 4. Conclusions

These results strongly suggest that the pharmacological induction of HO-1 combined with increased adiponectin levels in diabetic hypertensive rats alters the phenotype through a reduction in oxidative stress, thereby permitting endothelial cells to maintain an anti-apoptotic environment through increased expression of anti-apoptotic proteins and the restoration of endothelial responses thus preventing hypertension.
